# Consistency of Nutrition Recommendations for Foods Marketed to Children in the United States, 2009–2010

**DOI:** 10.5888/pcd10.130099

**Published:** 2013-09-26

**Authors:** Lorraine J. Weatherspoon, Elizabeth Taylor Quilliam, Hye-Jin Paek, Sookyong Kim, Sumathi Venkatesh, Julie Plasencia, Mira Lee, Nora J. Rifon

**Affiliations:** Author Affiliations: Elizabeth Taylor Quilliam, Sookyong Kim, Sumathi Venkatesh, Julie Plasencia, Nora J. Rifon, Michigan State University, East Lansing, Michigan; Hye-Jin Paek, Hanyang University, South Korea; Mira Lee, Chung-Ang University, South Korea.

## Abstract

**Introduction:**

Food marketing has emerged as an environmental factor that shapes children’s dietary behaviors. “Advergames,” or free online games designed to promote branded products, are an example of evolving food marketing tactics aimed at children. Our primary objective was to classify foods marketed to children (aged 2–11 y) in advergames as those meeting or not meeting nutrition recommendations of the US Department of Agriculture (USDA), Food & Drug Administration (FDA), Center for Science in the Public Interest (CSPI), and the Institute of Medicine (IOM). We document the consistency of classification of those foods across agency guidelines and offer policy recommendations.

**Methods:**

We used comScore Media Builder Metrix to identify 143 websites that marketed foods (n = 439) to children aged 2 to 11 years through advergames. Foods were classified on the basis of each of the 4 agency criteria. Food nutrient labels provided information on serving size, calories, micronutrients, and macronutrients.

**Results:**

The websites advertised 254 meals, 101 snacks, and 84 beverages. Proportions of meals and snacks meeting USDA and FDA recommendations were similarly low, with the exception of saturated fat in meals and sodium content in snacks. Inconsistency in recommendations was evidenced by only a small proportion of meals and fewer snacks meeting the recommendations of all the agencies per their guidelines. Beverage recommendations were also inconsistent across the 3 agencies that provide recommendations (USDA, IOM, and CSPI). Most (65%–95%) beverages advertised in advergames did not meet some of these recommendations.

**Conclusion:**

Our findings indicate that a large number of foods with low nutritional value are being marketed to children via advergames. A standardized system of food marketing guidance is needed to better inform the public about healthfulness of foods advertised to children.

## Introduction

In the United States, 1 in 3 children and adolescents aged 2 to 19 years is overweight (body mass index [BMI] ≥85th percentile) or obese (BMI ≥95th percentile) (1). Inappropriate high-calorie food choices and portions, relative to the amount of physical activity, contribute to childhood obesity (2). Other diet-related health considerations include consumption of total fat, saturated fat, added sugars, cholesterol, and sodium. These nutrients when consumed in excess can cause cardiovascular disease, hypertension, and other obesity-related conditions (3). Time spent using the computer, watching television, or using game consoles displaces the time and energy spent in playing sports and engaging in physical activity (4). Simultaneously, high-calorie foods such as sweetened beverages and French fries can lead to displacement of nutrients from more healthful foods (5). 

Food marketing has emerged as an environmental factor that shapes children’s dietary behaviors (6). “Advergames,” or free online games designed to promote branded products, are an example of evolving food marketing tactics aimed at children (7). Food advergames influence children’s food preferences and choices, and food products promoted in food advergames tend to have poor nutritional quality (8). Consumers often seek science-based nutrition guidelines for recognizing healthful foods from the US Department of Agriculture (USDA) (3), the Center for Food Safety and Applied Nutrition of the Food and Drug Administration (FDA) (9), the Center for Science in the Public Interest (CSPI) (10), and the food and nutrition board of the Institute of Medicine (IOM) (11). 

The objectives of this study were to 1) determine whether the foods marketed to children through advergames meet nutrition recommendations of the USDA, FDA, CSPI, and IOM and 2) examine the level of consistency across the 4 agencies by comparing food classification of the agencies. To our knowledge, no single federal regulatory agency or policy governs the nutrition content of foods marketed to children. Instead, the practice of self-regulation by food manufacturers allows the use of self-defined “science-based guidelines” rather than having a single source (12).

## Methods

We used comScore Media Builder Metrix (comScore, Inc, Reston, Virginia) to measure a webpage level audience database of 1.2 million Internet users from the United States. We identified food advergames visited by children aged 2 to 11 years from August 2009 through July 2010 that may have contained products from top-selling food companies, including those likely consumed by children. We obtained an extensive sample and comprehensive lists of the most recent food advergames from the top-selling food companies identified in previous studies (13), which was supplemented by the top 5 food brands identified in the Mintel Reports (14). A total of 475 URLs were identified. URLs were excluded if the website was not exclusively devoted to advergames or if the advergame was nonexistent (due to the changing nature of websites) or had other Web features such as product menu or videos. A total of 143 URLs met the criteria, including having at least 30 visitors per month (the visitor criterion set by comScore for tracking the URL), and were analyzed. We identified 439 foods from the 143 URLs, which resulted in an average of 3.06 unique foods per advergame (does not include repeated promotion of the same foods). Most of the brands represented in these advergames were promoted in children’s television programs (83.1%), and the food product categories were child-oriented (94.4%).

Two trained coders independently assessed the nutrient content of the 439 food products, representing 19 brands, identified in the advergames (average intercoder reliability = 0.93) (15). Coders were trained using an initial pilot sample of 143 foods. The nutritional content of the food products promoted in the advergames was calculated on the basis of nutrition facts labels displayed on food packages found either at the food company websites, grocery stores, or both.

We obtained information on serving sizes and total calories as well as information on the following: 1) total fat, 2) saturated fat, 3) added sugar, 4) sodium, and 5) cholesterol. The nutrition information obtained from the food packages was compared with the recommendations by USDA, FDA, CSPI, and IOM (Table 1) for the respective categories to determine if a food product met the recommendations for each of the 5 categories. To examine foods by meal categories, foods were also grouped by meal type: snacks (salty and sweet), meals (breakfast, lunch, and dinner), and beverages.

**Table 1:** Comparison of Guidelines for the Recognition of Nutrient-Dense Foods, by Agency, United States, 2009–2010

Nutrient	USDA Dietary Recommendations for Children Aged 2–11 Years	FDA Conditions for the Use of “Healthy”	CSPI Criteria for Foods Marketed to Children	IOM Tier I Nutrition Standards for Foods in School
**Calories**	1,000–2,200 daily	None	<1/3 RDA	≤200 kcal for snacks; ≤664 kcal for entrees
**Total fat**	<5% daily value; trans fats as low as possible	≤3 g per 100 g	≤35% kilocalories from fat	≤35% of kilocalories from fat/portion
**Saturated fat**	<10% of kilocalories	≤1 g per 100 g ≤10% of kilocalories from saturated fat	≤ 10% kilocalories from saturated fat plus trans fats	<10% of kilocalories from saturated fat; no trans fat
**Added sugar**	≤5% daily value	None	<35% added sugar	<35% of kilocalories/portion
**Sodium**	<1,500 mg daily; ≤5% daily value	≤600 mg for meals	≤230 mg for snacks; ≤770 mg for meals	≤200 mg for snacks; ≤480 mg for entrees
**Cholesterol**	<300 mg daily ≤5% daily value	≤90 mg/serving for entrees; ≤60 mg for all other foods	None	None

Abbreviations: USDA, US Department of Agriculture; FDA, US Food and Drug Administration; CSPI, Center for Science in the Public Interest; IOM, Institute of Medicine; RDA, Recommended Dietary Allowance.

For USDA, the dietary guidelines for children aged 2 to 11 years were chosen to compute the recommendations on the basis of a 2,000 kcal per day diet (3). The amounts of total fat, saturated fat, added sugar, sodium, and cholesterol in the food products were converted to percentage of calories obtained from each of these 5 categories, which were subsequently analyzed to determine whether they met the USDA recommendations. For all the other organizations, we used either the percentage of calories from the respective category when necessary or the cut points provided by the organizations for the different categories (Table 1).

## Results

Among the 439 foods from the 19 brands, there were 254 meals, 101 snacks, and 84 beverages (Table 2). Approximately 95% of the advertised meals and 78% of the snacks did not meet the USDA and FDA recommendations for total fat, and a large proportion of these foods met the recommendations of CSPI and IOM (only 13% of meals and snacks did not meet their fat recommendations). Similarly, nearly half of the snacks did not meet the saturated fat recommendations of USDA and FDA, while more than 80% of them met the CSPI and IOM saturated fat recommendations. However, less than one-fourth of the meals advertised met the USDA recommendations for saturated fat, while more than 65% of the meals met the recommendations of FDA, CSPI, and IOM.

**Table 2:** Proportion of Meals and Snacks Advertised Through Advergames That Met Agency Recommendations, by Agency, United States, 2009–2010

Nutrient	USDA n (%)		FDA n (%)		CSPI n (%)		IOM n (%)		Meets Standards of All Organizations
	Meets	Does Not Meet	Meets	Does Not Meet	Meets	Does Not Meet	Meets	Does Not Meet	
**Meals (n = 254)**									
Total fat	14 (5.5)	240 (94.5)	11 (4.3)	243 (95.7)	220 (86.6)	34 (13.4)	220 (86.6)	34 (13.4)	11 (4.3)
Saturated fat	58 (22.8)	196 (77.2)	167 (65.7)	87 (34.3)	188 (74.0)	66 (26.0)	183 (72.0)	71 (28.0)	58 (22.8)
Added sugar	34 (13.4)	220 (86.6)	NA		229 (90.2)	25 (9.8)	228 (89.8)	26 (10.2)	34 (13.4)^a^
Sodium	13 (5.1)	241 (94.9)	8 (3.1)	246 (96.9)	163 (64.2)	91 (35.8)	112 (44.1)	142 (55.9)	8 (3.1)
Cholesterol	230 (90.6)	24 (9.4)	253 (99.6)	1 (0.4)	NA		NA		230 (90.6)^b^
**Snacks (n = 101)**									
Total fat	22 (21.8)	79 (78.2)	22 (21.8)	79 (78.2)	88 (87.1)	13 (12.9)	88 (87.1)	13 (12.9)	22 (21.8)
Saturated fat	53 (52.5)	48 (47.5)	53 (52.5)	48 (47.5)	82 (81.2)	19 (18.8)	82 (81.2)	19 (18.8)	53 (52.5)
Added sugar	3 (3.0)	98 (97.0)	NA		27 (26.7)	74 (73.3)	26 (25.7)	75 (74.3)	3 (3.0)^a^
Sodium	60 (59.4)	41 (40.6)	46 (45.5)	55 (54.5)	98 (97.0)	3 (3.0)	92 (91.1)	9 (8.9)	46 (45.5)
Cholesterol	101 (100)	0	101 (100)	0	NA		NA		101 (100)^b^

Abbreviations: USDA, US Department of Agriculture; FDA, US Food and Drug Administration; CSPI, Center for Science in the Public Interest; IOM, Institute of Medicine; NA, not applicable.

a Meets recommendations of USDA, CSPI, and IOM.

c Meets recommendations of USDA and FDA.

Only a few foods met the USDA recommendations for added sugar (13.4% of meals and 3% of snacks), while 90% of meals and 26.7% and 25.7% of snacks met CSPI and IOM criteria, respectively. The FDA did not have recommendations for added sugar. The USDA and FDA had stringent recommendations for sodium; only 3% to 5% of meals and 46% (FDA) and 59% (USDA) of the snacks met the sodium recommendations. Conversely, a larger proportion of foods met the CSPI (64% meals and 97% snacks) and IOM (44% meals and 91% snacks) recommendations for sodium.

The CSPI and IOM did not have recommendations for cholesterol content of meals and snacks. Except for 1 meal, all met the recommendations for cholesterol according to FDA and 230 meals met the USDA recommendations (90%). All the advertised snacks met the USDA and FDA cholesterol recommendations. Overall, foods that met the USDA and FDA recommendations were consistent for total fat, cholesterol, saturated fat (only for snacks) and sodium (only for meals), while fewer foods met the CSPI and IOM recommendations.

Overall, meals only met the recommendations made by all 4 organizations for total fat (4%), saturated fat (23%), and sodium (3%). Thirteen percent of meals met the recommendations for added sugar by USDA, CSPI, and IOM, and 90% of the meals met the cholesterol recommendations by USDA and FDA. Conversely, 22%, 53%, and 46% of the snacks met the criteria for all the organizations for total fat, saturated fat, and sodium, respectively. Only 3% of the foods met the recommendations for added sugar by USDA, CSPI, and IOM, while all the foods met the cholesterol recommendations made by the USDA and FDA.

The nutrition recommendations made by USDA, CSPI, and IOM (FDA does not provide recommendations for beverages) for beverages that were examined in this analysis were also inconsistent (Table 3). Only the USDA and IOM recommend 100% fruit juice, and this was met by 10.7% of the beverages analyzed. The USDA and CSPI recommend no added sugars or sweeteners, and this criterion was met by 4 beverages analyzed for both agencies. The IOM recommends that beverages contain less than 22 g of sugar per 8 fluid ounces; 34.5% of beverages met this recommendation. CSPI is the only agency that mentions caffeine in its recommendations and recommends that beverages contain none; most (86.9%) beverages met this recommendation. The only recommendation that was consistent across all 3 agencies was for milk. All 3 recommend nonfat or 1% milk, but these beverage types were not found advertised through advergames.

**Table 3:** Proportion of Beverages Advertised Through Advergames That Met Recommendations of the USDA Dietary Guidelines for Americans, CSPI, and the IOM^a^, United States, 2009–2010

Beverage Recommendation (n = 84)	Meets Recommendation, n (%)	Does Not Meet Recommendation, n (%)
100% fruit juice^b^^,^ d	9 (10.7)	75 (89.3)
Nonfat and 1% milk^b^^,^ c ^,^ d	NA	NA
No added sugars or sweeteners^b^^,^ c	4 (4.8)	80 (95.2)
<22 g sugar per 8 fl oz^d^	29 (34.5)	55 (65.5)
Caffeine free^c^	73 (86.9)	11 (13.1)

Abbreviations: USDA, US Department of Agriculture; FDA, US Food and Drug Administration; CSPI, Center for Science in the Public Interest; IOM, Institute of Medicine; NA, not advertised.

a Food and Drug Administration (FDA) does provide recommendations for beverages.

b USDA recommendation.

c CSPI recommendation.

d IOM recommendation.

## Discussion

Our evidence indicates that the nutrition recommendations of the USDA, FDA, IOM, and CSPI for foods marketed to children vary greatly. Companies that market foods to children should exercise social responsibility, and clear criteria and enforcement of food advertising guidelines and regulations is warranted in the absence of consistent and enforceable voluntary standards.

The tactic addressed in this study, the advergame, is new, and the rapid pace of technological innovation will continue to provide more new and unique advertising opportunities for food companies. For example, the expanding popularity of mobile technology such as smartphones and tablets provides another venue for advertisers to reach children. In our technology-driven society, highly interactive advergames engage the player in ways that traditional one-way media, such as television, cannot. Food marketers, presumably recognizing the potential persuasive effect of advergames on children’s attitudes or behaviors, have used advergames to reach their target audience more frequently than have marketers of nonfood products (16). This reach is achieved by promotion of advergames on food product packages, on portal websites, and via television advertisements (17). As new techniques evolve, it becomes even more critical that parents and the public be provided with sound nutritional and health guidance through food marketers.

Advergames most often promote high-calorie food products (8,18), potentially blurring the lines between advertising and entertainment and compounding children’s inability to recognize and resist intent (19). Conversely, studies have also demonstrated that using advergames can be an effective method of increasing nutritious food choices (20) and can potentially be a positive alternative strategy for obesity interventions aimed at children.

Arguably, the food policy environment is complex, particularly within the context of food marketing to children; stakeholders representing industry, government, health policy, and consumer interests are often at odds over who should determine what marketers can and cannot do to sell their products. The food industry, advocating for self-regulation rather than government action, created the Children’s Food and Beverage Advertising Initiative (CFBAI) in 2006 (21). The original design of the program left the development of standards in the purview of each company, and, as a result of its self-regulatory nature, the CFBAI lacks strong compliance and enforcement powers (13). Five years later, the CFBAI announced the development of “uniform standards” to be effective December 13, 2013 (22). But even these new standards differ from those promulgated by the USDA, FDA, IOM, and CSPI, which are typically regarded as providing sound science-based guidance by consumers. Our findings call into question the ability of the consumer to make healthful choices when organizations they look to for guidance provide information that is not consistent.

In an effort to provide better information and help control and prevent childhood obesity and other diet-related health problems, a group of 4 federal agencies (the Federal Trade Commission, the Centers for Disease Control and Prevention, the FDA, and the USDA) was tasked with developing nutrition principles for foods marketed to children and adolescents ages 2 to 17 (23). This collaboration resulted in the Interagency Working Group on Foods Marketed to Children, formed in 2009, which proposed self-imposed guidelines that have not been implemented. These voluntary guidelines met with much resistance from the food industry and, at least at the time this study was conducted, the effort to adopt self-regulatory guidelines or enforce policy appears to be delayed indefinitely. The current state of self-regulation is ineffective (24), and inconsistent criteria from agencies result in a confusing environment for parents who are trying to provide their children with healthful food choices.

To our knowledge, this is the first study to use a data set of foods advertised through advergames that are actually reaching children and compare nutritional quality of foods in those games across nutrition recommendations from 4 organizations on which consumers rely for accurate nutrition information. The USDA provides the Dietary Guidelines for Americans for foods and beverages for people aged 2 years and older to facilitate a healthful diet. The FDA provides food labeling guidelines for the safety of the nation’s domestically produced and imported foods. The FDA also works in harmony with the Federal Trade Commission to establish guidelines for health and nutrition claims made in food marketing as well as on food labels. The IOM makes recommendations on appropriate nutrition standards for foods and beverages offered at schools, more specifically for those that are competing with federally reimbursable meals and snacks. The CSPI, a consumer advocacy organization, developed Guidelines for Responsible Food Marketing to Children intended for use by companies that market, sell or advertise foods to children less than 12 years of age.

Childhood obesity is a complex issue, and the food industry has the power of promoting foods that could positively affect children’s food preferences and consumption patterns (25). A multinational study of children aged 6 to 12 years in 10 countries (the United States, Australia, the United Kingdom, France, Germany, Denmark, Finland, Greece, the Netherlands, and Sweden) showed a positive association between the hours spent watching television advertisements promoting energy-dense (sweet, salty, or high-fat) foods and the prevalence of childhood overweight. This relationship was inverse when the advertisements promoted nutrient-dense foods (fruits, vegetables, bread, and fish products) (26). As mentioned in a review by Boyland and Halford, brand recognition as a result of various media exposure influences eating behaviors and food preferences of children (27). This result could also occur with advergames. Regulation of advertisement of foods high in calories, fat, salt, and sugar is imperative to protect the health of the younger generation.

Our study has limitations. Due to the nature of this study, it was not possible to demonstrate any actual effects of advergames on children’s dietary behaviors. And we could not ascertain whether multiple exposures from various media existed for any the food products identified. In addition, some food products appeared in an advergame multiple times, so the frequency of appearance may have been higher than reported and may be considered in future research. Finally, foods and meals that did not meet comScore’s criteria to generate audience information were excluded, even though they may have been used by and intended for children.

In our study, the classification of food categories varied across organizations due to varying criteria for nutritional quality developed to meet each agency’s purpose. Therefore, as postulated by Moodie et al, self-regulation or inconsistent regulation does not help adequately inform the public, and a uniform, preferably federal, policy should be established for guidelines for foods marketed to children (24). This policy should address the differences in what may be defined as “healthy” for foods marketed to children by industry versus nutrition experts.
